# The effectiveness of rural community health workers in improving health outcomes during the COVID-19 pandemic: a systematic review

**DOI:** 10.1080/16549716.2023.2292385

**Published:** 2024-01-05

**Authors:** Neema Kaseje, Meghna Ranganathan, Monica Magadi, Kevin Oria, Andy Haines

**Affiliations:** a London School of Hygiene & Tropical Medicine, London, UK; b Department of Research, Surgical Systems Research Group, Kisumu, Kenya; c Keele School of Medicine, Keele University Staffordshire, Newcastle-under-Lyme, UK; d Department of Research, Tropical Institute of Community Health, Kisumu, Kenya

**Keywords:** Rural, community health workers, COVID-19, pandemic preparedness, community based pandemic response

## Abstract

**Background:**

Rural community health workers [CHWs] play a critical role in improving health outcomes during non-pandemic times, but evidence on their effectiveness during the COVID-19 pandemic is limited. There is a need to focus on rural CHWs and rural health systems as they have limited material and human resources rendering them more vulnerable than urban health systems to severe disruptions during pandemics.

**Objectives:**

This systematic review aims to describe and appraise the current evidence on the effectiveness of rural CHWs in improving access to health services and health outcomes during the COVID-19 pandemic in low-and middle-income countries [LMICs].

**Methods:**

We searched electronic databases for articles published from 2020 to 2023 describing rural CHW interventions during the COVID-19 pandemic in LMICs. We extracted data on study characteristics, interventions, outcome measures, and main results. We conducted a narrative synthesis of key results.

**Results:**

Fifteen studies from 10 countries met our inclusion criteria. Most of the studies were from Asia [10 of 15 studies]. Study designs varied and included descriptive and analytical studies. The evidence suggested that rural CHW interventions led to increased household access to health services and may be effective in improving COVID-19 and non-COVID-19 health outcomes. Overall, however, the quality of evidence was poor due to methodological limitations; 14 of 15 studies had a high risk of bias.

**Conclusion:**

Rural CHWs may have improved access to health services and health outcomes during the COVID-19 pandemic in LMICs but more rigorous studies are needed during future pandemics to evaluate their effectiveness in improving health outcomes in different settings and to assess appropriate support required to ensure their impact at scale.

## Introduction

Globally, rural populations remain vulnerable to pandemics particularly in LMICs. As of November 2023, the current COVID-19 pandemic has led to 771 million infections and up to 18 million deaths have been attributed directly or indirectly to COVID-19 [1,2]. There are continued disparities in access to COVID-19 vaccines, COVID-19 therapeutics, and critical care capacity making the pandemic challenging to address, particularly in LMICs with significant rural populations [3–5]. Given the ongoing threat of current and future pandemics, evaluating key resources within rural health systems that can be deployed effectively to strengthen pandemic preparedness and response is vital.

Community Health Workers [CHWs] have been shown to be critical in global efforts to achieve Sustainable Development Goals [SDGs] and Universal Health Coverage [UHC] by 2030 [6]. CHWs were considered the cornerstone of primary health care in the 1978 Alma-Ata Declaration [1]. There is evidence to support CHW effectiveness in improving health outcomes during non-pandemic times, particularly in LMICs. A World Health Organization [WHO] systematic review of existing reviews showed that CHW interventions in LMICs were linked to improved physical activity, reduced repeated adolescent births, and reduced maternal, perinatal, and neonatal mortality rates [7]. Furthermore, a recent systematic review of CHW interventions demonstrated CHW effectiveness in improving population-based HIV-related health outcomes in LMICs [8].

There is some evidence that CHWs have also played an important role during the COVID-19 pandemic, especially in LMICs. A recent qualitative study found that CHWs made significant contributions in COVID-19 surveillance, community education, and support of those affected by COVID-19 in India, Bangladesh, Pakistan, Sierra Leone, Kenya, and Ethiopia [9]. These findings align with those of Bhaumik et al. who found that CHWs played a critical role during pandemics by participating in community engagement and contact tracing activities [10]. In addition, these findings are consistent with the WHO Strategic Preparedness and Response Plan which emphasises the need to listen to communities to reduce demand side barriers to health service utilisation and access during the COVID-19 pandemic [11].

Although these studies establish the important role CHWs played during the COVID-19 pandemic, they do not have a specific focus on rural CHWs and rural health systems in LMICs. There is a need to pay special attention to rural CHWs and rural health systems because they face more challenges compared to their counterparts in urban settings. Rural health systems frequently experience inadequate infrastructure, equipment, and consumables, and they have a more limited health workforce than in urban settings [12–18]. Globally, 75% of physicians and 65% of nurses work in urban areas [19]. In the US for instance, there are 30.8 physicians per 10,000 people in urban areas in contrast to 10.9 physicians per 10,000 people in rural areas [19]. And in terms of financing, rural health systems are facing financial crises resulting in hospital closures including in HICs [20]. As a result, compared to urban health systems, rural health systems have a reduced capacity to absorb shocks during pandemics and are more vulnerable to health system disruptions during pandemics including the COVID-19 pandemic. Furthermore, recent evidence suggests that during the COVID-19 pandemic, rural health systems were less prepared compared to urban health systems and COVID-19 responses were not adequately tailored to rural areas [21]. The findings argue for more evidence to be generated to guide rural pandemic preparedness and response efforts to mitigate the lack of preparedness during future pandemics. Moreover, there is growing and compelling evidence that the COVID-19 pandemic led to reduced access to health services making urgent the need to identify health interventions in rural health systems that can mitigate the negative impact of reduced access to health services during a pandemic. A systematic review of 81 studies from 20 countries found that the utilisation of diagnostic services, routine vaccinations, and surgical services decreased by a third during the COVID-19 pandemic [22]. Furthermore, more recent evidence shows significant reductions in the use of maternal and child health [MCH] services during the COVID-19 pandemic [23–26].

The objective of this systematic review is to describe and appraise the evidence of the effectiveness of rural CHWs in improving access to rural health services and subsequent rural health outcomes in LMICs during the COVID-19 pandemic with an intention to apply findings to future pandemics and outbreaks.

## Methods

### Search strategy

We conducted our searches in April and November 2023. We searched electronic databases, including PubMed/MEDLINE, EMBASE, Web of Science, WHO Global Health Library, and grey literature [Google Scholar, Clinical/Trials.gov, and the WHO International Clinical Trials Registry]. Searches identified articles that describe rural CHW interventions during the COVID-19 pandemic published from 2020 to November 2023. Our search terms used a combination of key terms: rural, and/or community health worker/primary healthcare worker/volunteer health worker/village health worker, and/or risk communication, and/or community empowerment, and/or pandemic, and/or COVID-19. Please see Table 1 for definitions of the different terms used in the paper.

**Table 1.** Definitions of terms.

Term	Definition
Community Health Workers	Refer to health workers working in communities. Depending on the country and the health system, they may be referred to as village health workers, volunteer health workers, lay health workers, and accredited social health activists [ASHAs] [27]
Rural areas	Refer to regions with population densities of fewer than 150 per square kilometer according to the OECD definition [28]
Health outcomes	A change in the health of an individual, group of people or population which is attributable to an intervention or series of interventions [29]
Low-and middle-income countries	Low income economies: Gross national income [GNI] per capita: $1,135 or less Lower middle income economies: GNI per capita: $1,136 to $4,465 Upper-Middle-Income: GNI per capita: $4,466 to $13,845 [30]

#### Conceptual framework for CHW effectiveness

**Figure 1.**
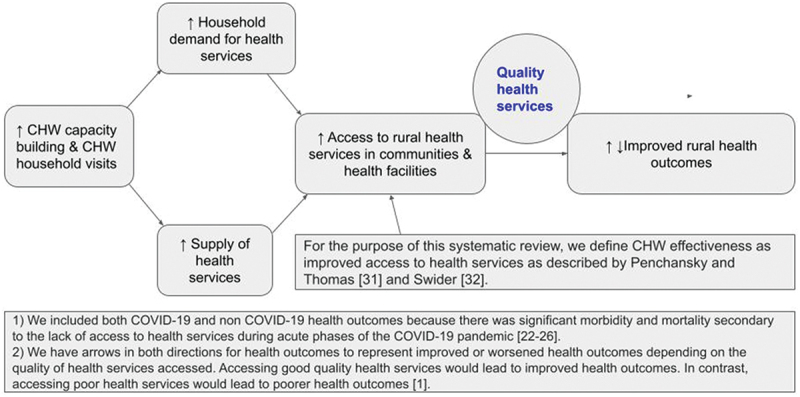
Conceptual framework [1,22–26,31,32]].

For the purpose of this systematic review, we define CHW effectiveness as improved access to health services as described by Penchansky and Thomas [31] and Swider [32] and improved downstream COVID-19 and non-COVID-19 health outcomes linked to CHWs visiting households to increase the demand for and the supply of health services during the COVID-19 pandemic in rural LMICs [Figure 1]. We included both COVID-19 and non-COVID-19 health outcomes because there was significant morbidity and mortality secondary to the lack of access to health services during acute phases of the COVID-19 pandemic [22–26].

### Eligibility criteria

We used the following inclusion and exclusion criteria:

- *Inclusion criteria*. We included experimental, non-experimental, quantitative and qualitative research that examined the effectiveness of CHWs during the COVID-19 pandemic in rural areas in LMICs.
- *Exclusion criteria*. We excluded opinion articles and commentaries that presented expert opinions but no original data, studies set in urban areas, and literature reviews/systematic reviews that addressed CHW interventions but did not specifically address rural CHWs during the COVID-19 pandemic. We used their reference lists, however, to find potential articles relevant to our systematic review. We excluded studies conducted in HICs.

Two reviewers [NK and MM] screened all articles independently by title and abstract and subsequently the full texts to determine whether articles under consideration met inclusion criteria. Any selection discrepancies were discussed by NK and MM to reach consensus.

We followed PRISMA reporting guidelines and presented results of the study selection process using the PRISMA 2009 Flow Diagram. We registered our review in the International Prospective Register of Systematic Reviews [PROSPERO registration number: CRD42022336485].

### Data extraction

**Figure 2.**
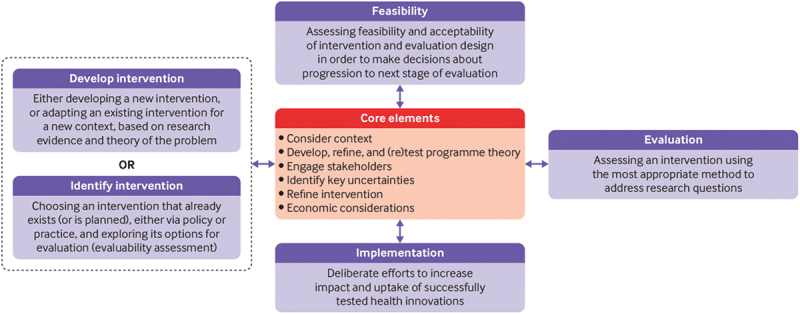
MRC complex interventions framework.

Once we established the list of included articles, NK independently exported study records to an Excel sheet, removed duplicate studies, and extracted data on study locations, publication years, study designs, interventions, outcome measures, main results, and intervention phases according to dimensions of the Medical Research Council [MRC] complex interventions framework (Figure 2 and Table 2). The MRC complex interventions framework was created to harmonise the evaluation of complex health interventions [33]. We used the most recent version of the MRC complex intervention framework to determine phases of CHW interventions in included articles. Following data extraction by NK, each data point was checked by MM.

**Table 2.** 

	Author/Year	Country	Study design	Objective	Intervention	Phases of community health worker interventions according to the MRC complex intervention framework: development, feasibility, implementation, and evaluation phases [33].	Outcome measures	Main results	Risk of bias (L= low, H= high)	Comparative component in the study design (Y=there is a comparative component in the research design; *N*= there is no comparative component in the research design)
35	Reddy KP, 2021	South Africa	Cost effective analysis	To develop a dynamic COVID-19 microsimulation model to assess clinical and economic outcomes and cost-effectiveness of epidemic control strategies in KwaZulu-Natal province, South Africa.	Modeling interventions included community health worker-led mass symptom screening	Development phase	Incremental cost-effectiveness ratio (ICER)	Incremental cost-effectiveness ratio = $340 per year of life saved	L	Y
36	Hernandez S, 2020	Guatemala	Pilot interventional study	To train community health workers in mitigating infection risk using a low literacy checklist while providing essential healthcare, such as prenatal care, during the COVID‐19 pandemic.	Community health workers used a low literacy checklist to provide essential healthcare, such as prenatal care, during the COVID‐19 pandemic	Feasibility phase	Number of traditional birth attendants trained, and the number of training sessions	8 traditional birth attendants were trained during 5 training sessions	H	N
37	Reinders S, 2020	Peru	Mixed methods evaluation of a community health worker maternal and neonatal health program in Peru.	To explore indigenous communities’ responses to the COVID-19 pandemic and its consequences for maternal and neonatal health (MNH) care in the Peruvian Amazon	Community-based maternal and neonatal program with comprehensive supervision covering monthly meetings with community health workers (CHW), community leaders, and health facilities.	Implementation phase	Clusters of suspected COVID-19 cases; availability of COVID-19 test kits, training, and medical face masks; suspension of routine antenatal and postnatal services; and community health worker home visits.	There were no COVID-19 testing kits or medical face masks; antenatal and postnatal were suspended; and 2 out of 3 community health workers resumed their household visits.	H	N
38	Kaweenuttayanon N, 2021	Thailand	Interventional study	To form, train, and deploy COVID-19 surveillance teams, including village health workers to identify returnees from high-risk areas, encourage self-quarantine for 14 days, and monitor and report the development of any relevant COVID-19 symptoms	Training and deploying rural village health workers to identify and monitor returnees from high risk COVID-19 areas	Feasibility phase	Village health worker household visits; referrals of suspected cases of COVID-19, and the national incidence of COVID-19 cases.	Village health volunteers visited more than 14 million households during March and April 2020. Volunteers identified and monitored 809 911 returnees, and referred a total of 3346 symptomatic patients to hospitals by 13 July 2020. The countrywide number of new cases steadily declined from the peak on 22 March 2020 to reach less than 10 new cases per day by 27 April 2020	H	N
39	Isaac R, 2021	India	Interventional study with time series analysis of COVID-19 seroprevalence	To establish and evaluate a COVID-19 PCR-testing programme and conduct two COVID-19 seroprevalence surveys in the same community.	Establishing a COVID-19 PCR testing programme and conducting community based COVID-19 testing.	Feasibility phase	COVID-19 seroprevalence	The two seroprevalence surveys showed COVID-19 positivity rates of 2.2% in July/August 2020 and 22.0% in November 2020.	H	Y
40	Shaikh I, 2021	Pakistan	Pilot interventional study	Connecting women via lady health workers to access abortion, contraception, and other gynecological services during the COVID-19 pandemic.	A novel hybrid telemedicine-community accompaniment pilot to provide abortion services, contraception, and other gynecological consultations.	Feasibility phase	Number of women referred by lady health workers; and complete uterine evacuation and reports of adverse events following abortion services.	176 women were referred by lady health workers. 90% of the women accessing abortion services reported complete uterine evacuation. No serious adverse events were reported following abortion services.	H	N
41	Joshi U, 2022	India	Community health worker training program development including costs	To assess the costs of developing a digital program for training community health workers to deliver a psychological treatment for depression in a rural district of Madhya Pradesh, India.	No intervention.	Development phase	Cost of developing a digital community health worker program	The total cost of developing a digital community health worker program was 208,814 USD	H	N
42	Sivakumar T, 2023	India	Prospective interventional study	To examine the impact of incentivizing Accredited Social Health Activists on the outcome of persons with severe mental illness (SMI) during the COVID-19 pandemic.	Training Accredited Social Health Activists to identify persons with severe mental illness from their villages and refer them for treatment	Feasibility phase	Mental health disability and illness severity; work functioning, and self stigma	At one year follow-up, there were significant reductions in disability, illness severity, and self-stigma, and there was improved work performance.	H	Y
43	Singh SS, 2022	India	Interventional study	To implement a 1-day COVID-19 training programme for rural, unaccredited community health workers who had recently completed a community health education course from the National Institute of Open Schooling.	A one day COVID-19 training programme for rural, unaccredited community health workers	Feasibility phase	The number of community health workers completing the COVID-19 training program and the proportion of community health workers satisfied with the training program.	15 000 community health workers completed the COVID-19 training programme and 80% (81/102) were satisfied with the training.	H	N
44	Garg S, 2022	India	Program evaluation of a community health worker intervention	To assess the time use and payments of multipurpose community health workers for the various roles they play.	A well-established community health worker programme in India’s Chhattisgarh state with 71,000 multipurpose community health workers.	Implementation and evaluation phases	Time spent doing community health worker tasks; type of work done; and community health worker payments in relation to minimum wage.	Rural community health workers spent 25.3 hours per week on their community health worker tasks. Time-use was well balanced between roles of service-linkage, providing health education and curative care, COVID-19 related work and action on social determinants of health. The average payment earned was less than 60% of legal minimum wage.	H	N
45	Kharel R, 2022	Nepal	Pilot interventional study	To train female community health workers on the COVID-19 response.	Innovative training programme to rapidly equip female community health workers with knowledge on the COVID-19 response	Feasibility phase	The number of community health workers trained; and the mean pre and post intervention community health worker knowledge scores	300 community health workers were trained. The mean knowledge scores increased from 4.1 to 6.3 (t (105) = 7.8, *p* < 0.001)	H	Y
46	Akter F, 2022	Bangladesh	Mixed-methods interventional study	To assess the fidelity and explore the barriers and facilitators of the implementation of a community-based comprehensive social behavior communication intervention to increase community resilience through prevention, protection, and care for COVID-19.	A community-based comprehensive social behavior communication intervention to increase community resilience through prevention, protection, and care for COVID-19	Feasibility phase	Community support team knowledge	Knowledge about wearing mask, keeping social distance, washing hands and COVID-19 symptoms were high (on average more than 70%) among community support team members.	H	N
47	Gore M, 2022	India	Qualitative study of accredited social health activists in India	To describe accredited social health activists’ (ASHAs) work roles before and during the COVID-19 pandemic, explore the tasks ASHAs performed throughout the pandemic, and understand its effects on the evolving role of ASHAs.	ASHAs were trained online and in-person to respond to the COVID-19 pandemic.	Feasibility phase	Perspectives of accredited social health activists	COVID-19 activities increased the workload and health risks of accredited social health activists leading to increased stress levels experienced by accredited social health activists.	H	N
48	Kok MO, 2023	Uganda	Mixed-methods interventional study	To assess the functioning of a telehealth intervention that was set up to support community health workers during the COVID-19 pandemic.	3,500 Community Health Workers (CHW) were trained to identify, refer and care for potential COVID-19 cases. A call center staffed by health professionals supported CHWs in diagnosing and managing patients with COVID-19.	Feasibility phase	Number of community health worker calls to the call center and stakeholder perspectives	There were 35,553 community health worker calls to the call center. According to community health workers, there were no signs that people in their communities were suffering from severe health problems due to COVID-19. After experiencing Ebola outbreaks, they were skeptical about thedangers of COVID-19 infections.	H	N
49	Gebremeskel AT, 2023	Ethiopia	Qualitative study of a community health worker program in Ethiopia	To critically examine the multifaceted fragmentation challenges of Ethiopia’s Community Health Workers (CHWs) program to deliver optimal maternal newborn and child health services.	A community health worker program delivering maternal newborn and child health services in rural Ethiopia	Evaluation phase	Perspectives of stakeholders	Stakeholder perspectives highlighted significant fragmentation of different components of the community health worker intervention including financing, supplies, community health worker empowerment and coordination, and stakeholder engagement	H	N

### Quality assessment

To assess the quality of the evidence in the included studies, we used the Cochrane Systematic Review Quality Assessment tool to assess the risk of bias [34]. We scored each of the seven criteria against a three-point rating scale corresponding to a high, low, or unclear risk of bias. NK evaluated the risk of bias.

### Synthesis of evidence

We conducted a thematic analysis and organised results according to the characteristics of included studies, CHW interventions and outcome measures during the COVID-19 pandemic, reported effectiveness of CHW interventions, and where available we reported stakeholder perspectives. In addition, we summarised the quality of the evidence and MRC phases of CHW interventions of included studies. We present our results in narrative and table forms.

## Results

**Figure 3.**
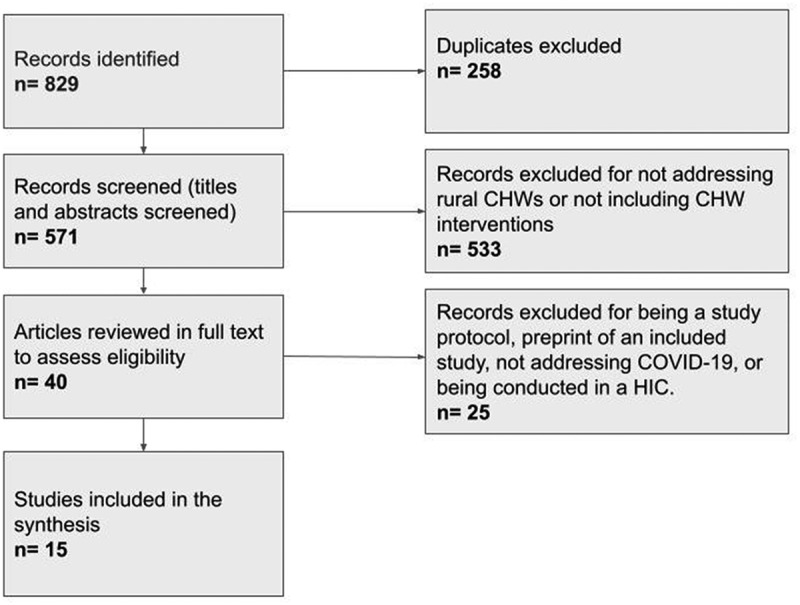
The PRISMA flow chart.

We identified 829 articles through electronic database searches; 571 articles remained following the removal of duplicates. NK and MM screened titles and abstracts of the 571 articles and excluded 533 articles as the focus was not on rural CHWs and/or did not include CHW interventions. We assessed the full texts of the remaining 40 articles for eligibility, and 25 articles were excluded for not addressing COVID-19 and/or being conducted in a HIC. In addition, two articles were study protocols; and a second article was a preprint of an included study. Fifteen articles met our inclusion criteria and were included in our analyses. Figure 3 of the PRISMA flow chart outlines the screening and study selection process.

Table 2 is for a summary of data extracted from the 15 included articles. We extracted data on study location, publication year, study design, objective, intervention, outcome measure/s, main results, phases according to the MRC complex interventions framework, and the quality of the evidence. In addition, we report on the risk of bias, and whether the study design had a comparative component.

### Characteristics of included studies

The 15 rural studies included in our systematic review were published from 2020 to 2023 and were from ten countries: South Africa [1], Uganda [1], Ethiopia [1], Guatemala [1], Peru [1], Thailand [1], India [6], Pakistan [1], Nepal [1], and Bangladesh [1,3,35–48]. Most studies were from Asia [10 of the 15 studies]; three studies were from sub-Saharan Africa; two were from the Americas [3,35,48].

There was a cost-effectiveness study [35] and interventional studies [36,38–42,44,45,47]. In addition, there were mixed-methods studies [37,46,48] and qualitative assessments of rural CHW interventions in India and Ethiopia [3,47].

### CHW interventions and outcome measures during the COVID-19 pandemic

CHW interventions were heterogeneous across the 15 studies. Interventions included a low literacy checklist to maintain access to prenatal care during the COVID-19 pandemic in Guatemala and CHW training in COVID-19 in Thailand, India, Nepal [36,38,42,44,47]. There were CHW interventions that leveraged previously established CHW programmes to respond to the COVID-19 pandemic in hard-to-reach communities in Peru and India [37,43]. Other CHW interventions sought to expand COVID-19 testing in India and strengthen linkages to abortion and mental health services during the COVID-19 pandemic in Pakistan and India [39,40,42]. In addition, rural CHWs strengthened COVID-19 prevention by influencing health behaviour in rural Bangladesh [46]. CHWs were also deployed to identify and refer possible cases of COVID-19 in rural Thailand, and in rural Uganda a call centre was established to support rural CHWs in community-based COVID-19 interventions [38,48].

In line with differences in rural CHW interventions, outcome measures were heterogeneous across the 15 studies. The outcome measures included: those related to CHW training, COVID-19 health outcome measures, non-COVID-19 health outcome measures, economic evaluation outcome measures [specifically the incremental cost-effectiveness ratio [ICER]] and stakeholder perspectives.

CHW training outcome measures included the number of participants trained and CHW satisfaction. There was a wide range in the number of participants trained: eight traditional birth attendants [TBAs] were trained in Guatemala [36]. The highest number of CHW participants was in India: 15000 CHWs completed their training in Bihar and 80% of those surveyed were satisfied with the training [43]. In addition, CHW COVID-19 knowledge was measured in Nepal, and the mean CHW knowledge score of 300 CHWs trained increased significantly from 4.1 to 6.3 [*p* < 0.001]; the maximum possible score was 10 [45]. In Bangladesh, more than 70% of community support team [CST] members including CHWs had increased knowledge of mask wearing, keeping social distance, and washing hands [46].

Four studies reported on COVID-19 specific outcomes including the incidence of COVID-19, COVID-19 community seroprevalence, and COVID-19 vaccine uptake. Reinders et al. reported clusters of COVID-19 cases among indigenous populations in the Peruvian Amazon but specific numbers of cases were not available at the time of publication [37]. Kaweenuttayanon et al. reported a significant drop in the daily number of COVID-19 cases to less than ten cases per day nationally following the CHW intervention in rural Thailand [38]. Isaac et al. in a community-based testing intervention documented the rise in COVID-19 seroprevalence by a factor of 10, as the pandemic progressed with rising community transmission [39]; a major limitation of this study was the absence of a comparison group without intervention that limited an assessment of the effectiveness of the CHW COVID-19 testing programme.

Three studies reported non-COVID-19 health outcome measures. Shaikh et al. reported on abortion outcomes during the COVID-19 pandemic in Pakistan [40]. Sivakumar et al. reported on disability from mental illness, mental illness severity and self-induced stigma in rural India during the COVID-19 pandemic [42].

Lastly, two studies had economic measures: Reddy et al. in a modelling study found that the ICER for an intervention including CHWs was $340 per year life saved; another study by Joshi et al. reported that the cost of developing a digital CHW programme was US$ 208,814 [35,41].

### The effectiveness of rural CHWs during the COVID-19 pandemic

**Figure 4.**
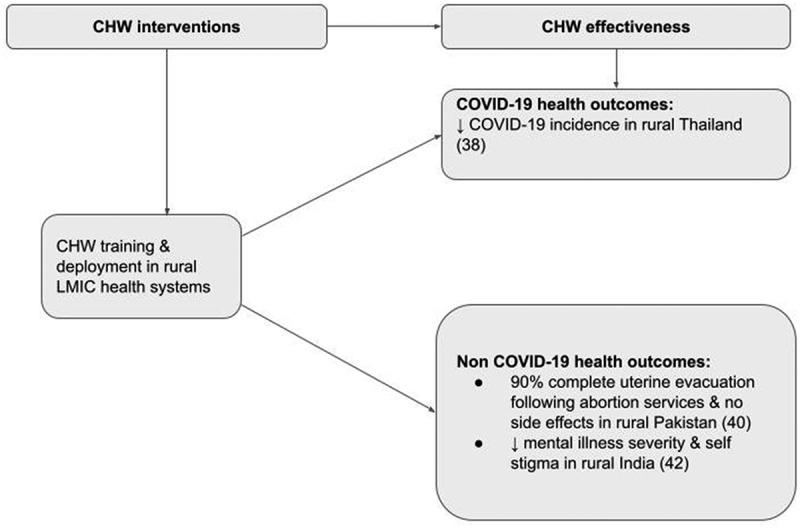
The effectiveness of rural CHWs in LMICs during the COVID-19 pandemic as shown by increased access to health services and improved COVID-19 and non COVID-19 health outcomes.

Three studies provided evidence on the effectiveness of rural CHWs during the COVID-19 pandemic by demonstrating increased access to COVID-19 and non-COVID-19 health services and improving individual and population health outcomes (Figure 4). Rural CHWs were effective in conducting household visits and referrals in Thailand: CHWs visited more than 14 million households from March to April 2020; they identified and monitored 809,911 returnees to rural Thailand and referred 3346 symptomatic patients to hospitals [38]. This CHW intervention was linked to a reduction in the incidence of COVID-19 cases in Thailand, from a peak of 188 cases per day to less than 10 cases per day during the early phases of the COVID-19 pandemic in March and April 2020 [38]. In Pakistan, 176 women were referred by CHWs for telehealth consultations to get abortion services [40]. As a result of this intervention, 90% of women reported complete uterine evacuation, and none reported side effects from accessing abortion services [40]. In India, mental health outcomes improved after continued linkage to mental health services through rural CHWs during the COVID-19 pandemic. As a result of this rural CHW intervention, there were statistically significant improvements in disability from mental illness, mental illness severity, and self-stigma due to mental illness compared to baseline measures: the mean WHO Disability Assessment Schedule 2.0 score was reduced from 16/100 at baseline to 12/100 at the second follow-up visit [*p* = 0.001] [42]. Because of the heterogeneity in outcome measures across studies, a pooled analysis of effect measures was not possible.

### Stakeholder perspectives

Five studies reported stakeholder perspectives. Stakeholders included CHWs, programme implementers, and programme evaluators. Stakeholder perspectives were diverse and highlighted concerns about COVID-19 vaccine misinformation, lack of PPE and testing kits, increased rural CHW workload and vulnerability to COVID-19 infection, and the suspension of antenatal and postnatal visits during the COVID-19 pandemic [37,47]. In Bangladesh, poor CHW training was seen as a hindrance to CHW effectiveness during the COVID-19 pandemic by CHWs [46]. In Uganda, in rural communities that had experienced Ebola outbreaks, CHWs felt there were no signs that people in their communities were suffering from severe health problems due to COVID-19 [48]. They felt COVID-19 symptoms were less severe and in sharp contrast to the severe symptoms seen in Ebola patients [48]. CHWs in rural Uganda also found that their community members were afraid to report symptoms, and they were afraid of being tested because they feared being quarantined and stigmatised [48]. With the telehealth intervention in rural Uganda, CHWs felt less isolated; contact with the call centre allowed them to provide better care, and it improved the supply of medicine and other essential health products [48]. In Ethiopia, a qualitative study on a CHW intervention designed to deliver maternal, newborn, and child health in rural Ethiopia demonstrated significant fragmentation of different components of the intervention including financing, supplies, CHW empowerment and coordination, and stakeholder engagement [3].

### Quality of the evidence

Overall, the quality of the evidence was poor: 14 out of the 15 studies had a high risk of bias. Sources of bias included reporting bias, recall bias, selection bias, and observation bias. There were no randomised controlled trials [RCTs]. Due to the high risk of bias, the chances of overestimating or underestimating the effectiveness of rural CHWs in improving health outcomes during the COVID-19 pandemic were high. Furthermore, the causal link between rural CHW interventions and rural CHW effectiveness in improving COVID-19 and non-COVID-19-related health outcomes was weakened by the lack of comparative components in study designs. Only 4 out of 15 studies had comparative components in their research designs: the first study, a cost-effectiveness analysis, compared different combinations of five COVID-19 public health interventions including health-care testing alone, diagnostic testing at health care centres; contact tracing in households with cases; isolation centres for cases not requiring hospital admission; mass symptom screening with testing of symptomatic individuals by CHWs; and quarantine centres for household contacts who test negative [35]. The second study compared COVID-19 seropositivity rates across different time points [39]. And the remaining two studies compared pre- and post-intervention mental health outcome measures and CHW knowledge [42,45].

### Phases of CHW interventions according to the MRC complex intervention evaluation framework

We found that most studies addressing the effectiveness of rural CHWs in improving health outcomes during the COVID-19 pandemic were in feasibility and pilot phases of the MRC framework. Specifically, two studies were in the design and modelling phases [35,41]. Seven studies were in feasibility and pilot phases [36,38–40,42,43,45,45–48]. Three studies described well-established CHW programmes that were used to respond to the COVID-19 pandemic [3,37,44].

## Discussion

During pandemics and other shocks, rural CHWs face greater challenges because rural health systems are under-resourced compared to urban health systems. Therefore, a focused examination of their effectiveness during the COVID-19 pandemic is important. To our knowledge, this is the first review to examine the effectiveness of rural CHWs during the COVID-19 pandemic.

During the COVID-19 pandemic, rural CHW interventions were carried out in multiple regions, particularly in LMICs where health systems were experiencing critical gaps in resources. From the regional distribution of studies, we can infer that health systems with greater gaps in human resources were more likely to implement rural CHW interventions during the COVID-19 pandemic. This was to maximise prevention and delay the influx of a high number of severe COVID-19 cases that would rapidly overwhelm their health systems. The possibility that health systems would be rapidly overwhelmed was a significant concern in LMICs, particularly in SSA [49–51]. As a result, relative differences in approaches emerged early during the COVID-19 response depending on resources that were available. In HICs, there was a heavier focus on hospital care that was more readily available; and the management of severe COVID-19 cases frequently involved mechanical ventilation [52]. In contrast, in LMICs, there was an emphasis on community-based approaches. In rural Vietnam, Tran et al. described the benefits of deploying village health workers to strengthen community surveillance efforts by expanding the population coverage in a setting with low COVID-19 testing capacity [53]. In Kenya, where 70% of the population is rural, home-based care of COVID-19 patients was rolled out in July 2020 [4 months after the pandemic was declared]; and some rural counties, such as Siaya county built the capacity of CHWs to maximise COVID-19 prevention and optimise its case management at the community level [54,55]. In future pandemic preparedness and response strategies, integrated approaches with interventions implemented at community and health facility levels could be synergistic and are worth considering.

We observed differences in interventions and health outcomes reflecting differences in CHW roles across different settings during the COVID-19 pandemic. CHWs promoted COVID-19 prevention measures; they participated in the early detection and management of COVID-19 cases, and they sustained linkages to key essential health services during the COVID-19 pandemic with improved COVID-19 and non-COVID-19 health outcomes as previously described (Figure 4). Other studies have found improved disease-specific health outcomes following rural CHW interventions. For instance, in the case of dengue fever, an emerging pandemic, a study from Vietnam showed a dengue control efficacy rate of 99.7% following a rural CHW intervention [56]. Furthermore, in a Nicaraguan and Mexican randomised controlled trial, there was a 29.5% reduction in dengue infections in CHW intervention clusters [57].

During a pandemic, providing essential and comprehensive health services for a range of conditions is also important to prevent increased mortality from unrelated causes. A systematic review of 81 studies from 20 countries found that the utilisation of diagnostic services, routine vaccinations, and surgical services decreased by a third during the COVID-19 pandemic [22]. Furthermore, more recent evidence shows significant reductions in the use of maternal and child health [MCH] services during the COVID-19 pandemic [22–26]. Similar observations were made during the Ebola outbreak in Guinea, Sierra Leone, and Liberia where there were sharp reductions in the use of MCH services [58]. However, with CHW training and support, the use of MCH services rebounded [58]. These results align with our findings of improved non-COVID-19-related health outcomes following rural CHW interventions (Figure 4). By strengthening links to routine and comprehensive health services during pandemics, rural CHWs can mitigate significant reductions in the use of essential and comprehensive health services during pandemics. These findings support the inclusion of rural CHWs in pandemic preparedness and response strategies.

Stakeholder perspectives are particularly useful because they provide information on key gaps that should be addressed during future pandemic response efforts. Stakeholder perspectives varied across studies; however, key insights that emerged across regions were that: CHWs remained committed to delivering COVID-19 and non-COVID-19-related health services despite increasing workloads and fear of contracting COVID-19. This is consistent with the findings of a study from Rwanda [59]. Another overarching theme was the need for more rural CHW training. This finding aligns with a recent WHO systematic review that found that training was critical to CHW effectiveness [7]. In countries where access to vaccines was delayed – vaccine supply was also a significant concern [4]. In addition, we found limited qualitative data on attitudes, perceptions and experiences of CHWs represent a gap in the current evidence that should be addressed in future studies. Further understanding of CHW attitudes, perceptions, and experiences would provide important insights for future CHW interventions during pandemics.

The methodological limitations in research designs led to a high risk of bias from multiple sources. The early COVID-19 response was an emergency, and rapid action was favoured to save as many lives as possible. Because of these initial priorities, designing, piloting, implementing, reporting and evaluating interventions with well-designed impact assessments was challenging [60]. Moreover, during the initial phase of the COVID-19 pandemic, vaccines were not available, and the risk of contracting and potentially dying from COVID-19 was significant; this made clinical and research activities very challenging.

Our systematic review has several strengths. First, it focuses on rural CHWs who are more likely to experience lack of resources and support [61]. Second, our review demonstrates that it was feasible and effective to train rural CHWs during the COVID-19 pandemic. In addition, we show that deploying trained and supported rural CHWs appeared to lead to improved COVID-19 and non-COVID-19 health outcomes across regions, a finding which is consistent with the potentially critical role rural CHWs can play during pandemics. In addition, in contrast to other studies, our review examined phases of evaluation of CHW interventions that showed that most studies were in feasibility and pilot phases; highlighting a need for more consistent and sustained investments in building evidence around effective community-based interventions during pandemics.

There may however be evidence we did not capture in our search, for example because some reports are in the grey literature that were not captured by our search. Calculating a composite effect measure across different interventions was not possible because of the heterogeneity in study designs, interventions, and outcome measures. The majority of included studies had a high risk of bias and the lack of comparative components in study designs meant that conclusions were not definitive. Our findings are specific to the COVID-19 pandemic and may not fully apply to other pandemics.

For policy-makers with significant rural populations and limited resources, engaging rural CHWs is a potential solution for strengthening pandemic preparedness and response efforts using a cadre of health workers already in place. Our review provides some evidence that CHWs were able to effectively care for COVID-19 patients, and they also maintained linkages to essential and comprehensive health services during the COVID-19 pandemic.

Different response strategies to the COVID-19 pandemic emerged as the pandemic progressed; well-resourced health systems emphasised hospital care – and resource-constrained health systems tended to emphasise community-based approaches. Future policy action in pandemic preparedness and response should consider an integrated approach with interventions to strengthen both hospital care and community-based health care to maximise the potential number of lives that can be saved.

Stakeholder perspectives, although limited, provided key insights on current gaps in CHW interventions that need to be addressed including more CHW training and more CHW support with PPE, and other essential supplies. Better designed studies, which limit sources of bias and confounding factors, are needed to further explore the effectiveness of rural CHWs in improving health outcomes during pandemics. Randomised controlled trials [RCT] [most likely cluster RCTs] would be the gold standard but are difficult to undertake in emergency situations. Guidance on the evaluation of complex interventions should shape future research.

Furthermore, there is a need for cost-effectiveness data on rural CHW interventions during pandemics to help policy-makers make decisions on what interventions would be most effective when resources are limited. Additionally, we found a lack of mortality data in studies published to date. Mortality data would provide more compelling evidence on the effectiveness of rural CHWs in improving health outcomes during pandemics but will be increasingly difficult for COVID-19 as death rates have fallen. Lastly, more qualitative data would be useful to gain a better understanding of stakeholder perspectives to guide future action in pandemic preparedness and response.

## Conclusions

The current evidence suggests that rural CHWs may be effective in improving access to health services and health outcomes during the COVID-19 pandemic in rural LMICs but the quality of studies included in this evidence synthesis is poor. Given the threat of future pandemics, and the need to strengthen rural health system responses, there is a need for better designed studies to generate high-quality evidence on the effectiveness and cost-effectiveness of rural CHWs in improving health outcomes during pandemics.

## Paper context

Rural health systems face greater challenges in terms of having an adequate number of health care workers, infrastructure, and equipment which limit their capacity to respond to pandemics including the COVID-19 pandemic. Rural CHWs play an important role in improving health outcomes. Our systematic review findings suggest that rural CHWs improved health outcomes during the COVID-19 pandemic, and they are a potential critical resource to be leveraged to strengthen rural pandemic preparedness and response efforts.
